# Serological study on brucellosis in captive elephants (*Elephas maximus*) and stray dogs in North Thailand

**DOI:** 10.14202/vetworld.2020.1992-1997

**Published:** 2020-09-26

**Authors:** Suchanit Ngamkala, Taweepoke Angkawanish, Weerapun Nokkaew, Nikorn Thongtip

**Affiliations:** 1 Department of Veterinary Technology, Faculty of Veterinary Technology, Kasetsart University, Bangkok 10900, Thailand; 2 The National Elephant Institute, The Forest Industry Organization, Lampang 52190, Thailand; 3 Clinic for Wildlife, Faculty of Veterinary Medicine, Mahanakorn University of Technology, Bangkok 10530, Thailand; 4 Department of Large Animal and Wildlife Clinical Sciences, Faculty of Veterinary Medicine, Kasetsart University, Kamphaeng Saen Campus, Nakhon Pathom 73140, Thailand; 5 Center for Agricultural Biotechnology, Kasetsart University, Kamphaeng Saen Campus, Nakhon Pathom 73140, Thailand; 6 Center of Excellence on Agricultural Biotechnology: (AG-BIO/PERDO-CHE), Bangkok 10900, Thailand

**Keywords:** Asian elephants, brucellosis, serology, stray dogs

## Abstract

**Background and Aim:**

Brucellosis is considered as an important zoonotic disease caused by various strains of *Brucella* in numerous host species. Although brucellosis has been reported in almost animal species, the relevance of brucellosis infection and diagnostic technique in Asian elephant (*Elephas maximus*) has been limited. The present serological investigation aimed to investigate the antibody response to *Brucella abortus* in captive Asian elephants in North Thailand. Moreover, further serological survey was also conducted to detect the antibody response to *Brucella canis* in stray dogs cohabiting the same area as the elephant herd.

**Materials and Methods:**

Serum samples were collected from 40 captive Asian elephants and submitted for serological analysis based on *B. abortus* antigen using Rose Bengal plate test (RBPT) in combination with ethylenediaminetetraacetic acid-tube agglutination test (EDTA-TAT) as a supplementary test and by commercial indirect enzyme-linked immunosorbent assay (iELISA). In addition, serum samples were also obtained from 16 stray dogs that live nearby the elephant-raising area and were tested using commercial Dot-ELISA based on *B. canis* antigen.

**Results:**

Serological analysis in captive Asian elephants showed 100% seronegative (40/40) from all serological tests response to *B. abortus*. For stray dogs, 12.5% (2/16) had a low positive reaction response to *B. canis*.

**Conclusion:**

The serological survey for brucellosis in Asian elephant was adapted and applied using RBPT, EDTA-TAT, and iELISA in the present study. For future evaluation, we recommended the use of a combination of serological tests with validation together with comparing by direct detection such as bacterial isolation to provide an appropriate brucellosis surveillance program in Asian elephants. In addition, the surveillance of stray dogs or multispecies habitation should be kept into considerations.

## Introduction

The Asian elephant (*Elephas maximus*) has been listed as an endangered species in the Appendix I of the Convention on International Trade in Endangered Species of Wild Fauna and Flora. In Thailand, the elephant population has declined over the years due to poor management approaches, diseases, and human-elephant conflicts, such as poaching and habitat loss from expansion of human settlements and agricultural fields [1,2]. Thailand has a large number of captive elephants used in tourism industry and for work activities by private owners. However, sustainability problems occur in captive populations as well, where reproduction rate is low [3]. It is critical to conserve and increase the populations of Asian elephants using both natural breeding [4] and assisted reproductive technology [5]. Moreover, expanding the elephant population should be performed with good husbandry and health management, specifically monitoring of infectious diseases on the reproductive system. An important infectious disease causing reproductive disorder in domestic and wild animals includes brucellosis, which has significant zoonotic potential. The disease can result in infertility, abortion, retained placenta, stillbirths, reproductive organ inflammation, and other reproductive disorders with significant economic repercussions [6-8].

Brucellosis is caused by *Brucella*, Gram-negative, non-motile, non-spore-forming, aerobic, facultative intracellular coccobacilli. It can transmit across species [9]. Despite their various host preferences and broad distribution, *Brucella abortus* and *Brucella suis* have been majorly isolated from several terrestrial wildlife species, such as camel, bison, elk, African buffalo, wild boar, red fox, and reindeer [6,8,10-12]. However, *Brucella melitensis* is rarely reported in wildlife. Interestingly, *Brucella canis*, which is responsible for canine brucellosis, has been reported in wild canid with limited importance in wildlife [6]. Since brucellosis surveillance programs in several countries, including Thailand, mainly focus on *B. abortus*, which is responsible for bovine brucellosis, the identification of possible infection of *B. abortus* in wildlife, including Asian elephants, has been paid careful attention. Nevertheless, our understanding of the persistence of *Brucella* spp. in Asian elephants is limited. Moreover, an important risk factor of wildlife brucellosis is *Brucella* transmission among multiple host species in the same environment or raising system. Hence, wildlife brucellosis must be considered and investigated as a potential reservoir for implementing control and monitoring system to decrease *Brucella* infections in wildlife populations [13].

Laboratory diagnosis of brucellosis includes direct methods involving bacteriological analysis or molecular identification and typing based on the detection of specific sequences of *Brucella* spp. and indirect methods applying serology for examination of specific antibody level after *Brucella* spp. infection [14]. According to the World Organization for Animal Health, formerly the Office International des Epizooties (OIE), the serological examinations for wildlife brucellosis are important and generally performed for screening purposes [8]. Interestingly, most wildlife brucellosis serology are usually achieved using the same antigens as in domestic ruminant analysis and have been directly transposed to wild species from their use in domestic livestock populations without any previous validation [6]. Therefore, the serological survey for brucellosis in captive Asian elephant herd in Thailand and other animals cohabiting the same area as the elephant herd should be investigated, contributing to the eradication of policies and studying of epidemiological situation.

Therefore, the present study aimed to investigate and conduct serological surveys of the antibody response to *B. abortus* in the herd of captive Asian elephants in North Thailand together with the antibody response to *B. canis* in stray dogs cohabiting in and around the same areas of the Asian elephant herd.

## Materials and Methods

### Ethical approval

This study was approved by the Kasetsart University Institutional Animal Care and Use Committee in accordance with university regulations and policies governing the care and use of laboratory animals (ACKU63-VTN-001).

### Animals, sample size, and study area

The investigation was conducted in the elephant camp in North Thailand between August and December 2019. The study randomly selected 40 captive Asian elephants (*E. maximus*) (17 males and 23 females) aged between 4 and 65 years (average, 28.03 years) during routine disease surveillance activities by the camp. In general, all elephants showed normal general appearance, except for 2 female elephants (8.7%) with a clinical history of repeated breeding and late-term abortion. Furthermore, the investigation was performed, and 16 healthy stray dogs (*Canis familiaris*) cohabiting nearby the elephant-raising area were randomly selected and included in the preliminary survey. The sample size of captive Asian elephants and stray dogs in this study (estimating proportions) was determined, according to Daniel and Cross [15].

### Sample collection

Venous blood samples were obtained aseptically from the auricular veins in captive Asian elephants (10 mL) (n = 40) and from the cephalic or lateral saphenous veins in stray dogs (3-5 mL) (n = 16), were allowed to clot at 37°C for 30 min, and subsequently centrifuged at 3,500× *g* for 10 min to obtain the serum. Subsequently, serum separation was processed in a biosafety laboratory (Biosafety Level 2 enhanced) and stored at −20°C before serological analysis.

### Serological test

The serological analysis for *Brucella* infection in terrestrial animals was performed based on the examination standard recommended by the Thailand National Animal disease surveillance system and the World Organization for Animal Health [8]. The serum samples of captive Asian elephants were evaluated for antibody response to *B. abortus* and were assessed using the buffered *Brucella* antigen test or Rose Bengal plate test (RBPT) in combination with ethylenediaminetetraacetic acid-tube agglutination test (EDTA-TAT) as a supplementary test and commercial indirect enzyme-linked immunosorbent assay (iELISA) [6,16]. Moreover, the serological analysis in stray dogs was also examined for antibody response to *B. canis* using commercial Dot-ELISA. All serological procedures were performed in the biosafety laboratory (Biosafety Level 2 enhanced). Moreover, the used samples and all equipment were disinfected in 1-6% sodium hypochlorite solution (Clorox Co., USA) for at least 2 h and were subsequently autoclaved at 121°C for 15 min before discard.

### RBPT and EDTA-TAT in captive Asian elephants

The use of agglutination tests was employed for the diagnosis of many diseases in Asian elephants [17-19] and other wildlife [20]. In the present study, the cell suspensions of *B. abortus* antigen for RBPT and EDTA-TAT, the positive and negative reference controls, were provided by the National Institute of Animal Health, Thailand, and performed following the procedure described by OIE [8]. Briefly, the RBPT was achieved by mixing 30 μL buffered antigen with 30 μL serum sample (or negative or positive reference controls) on the sterile plate and agitating gently for 4 min. Subsequently, the positive agglutination reaction was observed.

Moreover, the EDTA-TAT was applied for each serum sample. Briefly, *B. abortus* antigen was first diluted for 1:100 using 10 mM EDTA-phosphate-buffered saline solution (pH, 7.2). Next, 2 mL of the diluted antigen was added into tube no. 1, while 1 mL of that antigen was added into tube no. 2-5 followed by mixing 80 μL of serum sample into glass tube no. 1. Subsequently, 1 mL of the mixed solution from glass tube no. 1 was transferred to glass tube no. 2 (this process was repeated to each glass tube). Moreover, 1 mL of the solution in glass tube no. 5 was discarded to make successive 2-fold dilutions. Finally, serum concentrations in glass tube no. 1-5 were 1:25, 1:50, 1:100, 1:200, and 1:400, respectively. After a slight agitation, all tubes were incubated at 37°C for at least 48 h and observed for the agglutination reaction. For interpretation, total agglutination at 1:100 or greater was recognized as a positive result, whereas no agglutination or that <1:50 was considered as a negative result.

### iELISA in captive Asian elephants

iELISA was employed using PrioCHECK^®^ Brucella Ab kit (Prionics AG^©^, Netherlands) for detecting serum-specific immunoglobulin G1 (IgG1) antibody against *B. abortus* and *B. melitensis*. A microtiter plate was first coated with sonicated and inactivated *B. abortus* antigen. Serum samples were dispensed and incubated in the coated wells of a microtiter plate. The bound antibodies were detected using an anti-IgG monoclonal antibody. All steps of the iELISA were conducted with the manufacturers’ protocols. Optical density of each sample was measured at 450 nm within 15 min and calculated as percent positivity (PP) relative to the positive control. PP values <45% were recognized as a negative result, whereas PP values >45% were recognized as a positive result.

### Dot-enzyme-linked immunosorbent assay in stray dogs

According to Mol *et al*. [21] for the detection of canine brucellosis, commercial Dot-ELISA was conducted using the ImmunoComb Canine Brucella Antibody Test Kit^®^ (Biogal-Galed Laboratories, Israel) to detect *B. canis*-specific antibodies (IgG) in the serum of stray dogs. All procedures of the Dot-ELISA were performed using the protocol outlined by the manufacturer. At the end of the examinations, a purple-gray color was developed in all positive reference spots and in positive samples. The color intensity was dependent on antibody level. Results were scored using the positive reference spot and CombScale score reading provided by the kit. Moreover, a color tone that was equal or darker than the reference spot was considered a positive response. Color fainter than the positive reference indicated a low response. Thus, the positive result was graded as high, medium, or low reactions against *B. canis*.

## Results

### RBPT, EDTA-TAT, and iELISA in captive Asian elephants

All serum samples were 100% seronegative (40/40) for specific antibodies against *Brucella* infection examined by RBPT, EDTA-TAT, and iELISA. The results indicated no or low exposure to *B. abortus* (smooth *Brucella* spp.) in captive Asian elephants in North Thailand.

### iELISA in stray dogs

Serum samples were 12.5% seropositive with low positive reaction (2/16) for antibodies against *B. canis* examined. The result indicated low exposure to *B. canis* in some stray dogs.

## Discussion

Over the years, *Brucella* infection had varied among geographic areas [22], which was documented in domestic animals and wild species worldwide. Despite the previous reports demonstrating that the main *Brucella* spp. isolated from wild animals were smooth *Brucella* spp. [6,23,24], the studies on wildlife brucellosis have been limited to some species, specifically in Asian elephants. Consequently, there are less information available for Asian elephant brucellosis. Therefore, this study aimed to establish the serological surveys focused on antibody response to *B. abortus* in captive Asian elephants from the elephant camp in North Thailand. The serological tests for Asian elephant were based on the detection of serum *Brucella*-specific antibody against *B. abortus* antigen, associated with the smooth lipopolysaccharide (sLPS). Importantly, sLPS is shared by all the naturally occurring biovars such as *B. abortus*, *B. melitensis*, and *B. suis* that had been identified for wildlife infection [6].

Serology is the most common test performed for animal brucellosis worldwide with validation. It is used for screening purposes to classify the possible exposure of the pathogens in different wild animals [11,25]. However, the validation of serological test capacity to precisely predict the elephant brucellosis can be difficult because it relies on the diagnostic sensitivity and diagnostic specificity parameters that are verified using brucellosis-free and truly infectious status in animals [26]. Unfortunately, there is no existing evidence regarding true *Brucella* infection in elephant elsewhere. Therefore, most serological tests in Asian elephants in this study have been directly applied with the same principles and antigens as in bovines without any validation [14]. The sensitivity and specificity of some serological tests for the diagnosis of cattle brucellosis as published in the literature are shown in Table-1 [14]. In addition, we used a combination of three serological tests to increase the diagnostic performance. The serum samples from 40 captive Asian elephants were examined for antibodies to *B. abortus* using the RBPT and EDTA-TAT with standardized *B. abortus* antigens. In addition, commercial iELISA, PrioCHECK^®^ Brucella Ab kit (Prionics AG^©^, Netherlands), was also employed. All three serological tests were performed essentially as for bovines as described by OIE [8].

**Table-1:** Sensitivity and specificity of serological methods for the detection of cattle brucellosis; modified from Godfroid *et al*. [14].

Serological methods	Sensitivity (%)	Specificity (%)
BAT	87.0	97.8
iELISA	97.2	97.1-99.8
SAT	81.5	98.9

BAT=Buffered *Brucella* antigen test, iELISA=Indirect enzyme-linked immunosorbent assay, SAT=Slow agglutination test

The RBPT can be recommended as a regular purpose diagnostic test in all wildlife species. There were reports on using RBPT for screening brucellosis in camel [27], elephants [28], and other wild animals [29,30] as the procedure was simple, rapid, and highly sensitive and specific, and it guarantees the absence of infection in brucellosis-free herds [8]. In addition, the previous studies of *Brucella* spp. infection were performed: Both RBPT for screening and serum TAT for confirmation that are utilized in serological surveys in various wild animals [20,31]. The TAT is one of the conventional serological tests based on the agglutination reaction that occurs slowly, referred to as slow agglutination test. Moreover, this technique could be used as a confirmatory test in wild animals [30]. In the present study, we used the TAT with minor modification by adding EDTA to significantly improve the specificity of the test [14,16]. Furthermore, detection of elephant brucellosis had been focused on iELISA, which is a diagnostic confirmation method with high specificity and sensitivity. Although the PrioCHECK^®^ Brucella Ab kit (Prionics AG^©^, Netherlands) was a commercial iELISA and originally developed and validated for use as a detection tool for antibodies against *B. abortus* antigens in bovine brucellosis, we adapted this kit as off-label use in the present study for serodiagnostics in Asian elephants. We hypothesized that anti-IgG monoclonal antibody provided in commercial iELISA kit might be possibly cross-reacting to the Asian elephant IgG against *B. abortus* antigen. In addition, a previous study revealed the potential cross-reactivity of Asian elephant IgG to anti-bovine IgG (65.3%) compared to the homologous Asian elephant IgG reaction [32]. It suggested the elephant IgG shared similar property to bovine IgG. Therefore, the present study showed 100% seronegative results from RBPT, EDTA-TAT, and commercial iELISA investigation, suggesting no or low exposure to *B. abortus* (smooth *Brucella* spp.) in captive Asian elephants in North Thailand. Notably, the possible interferences might occur due to non-validation immunoassay and caused false seronegativity. The interferences included limited affinity and specificity of the test antibody for elephant IgG, non-specific binding by undesired antibody activity or other cross-reactivity substances, and endogenous antibody in serum sample and serum quality [33]. Nevertheless, we considerably conducted the serological tests to minimize the negative interferences by serum quality control, such as avoiding of hemolysis or lipemia blood samples in the present study.

Importantly, to effectively control and provide the brucellosis surveillance in wildlife, we must initially identify the pathogen(s) in multiple host species that are possible epidemiological sources of infection within the same environment of wildlife herd. Therefore, we performed further examination of the serological survey for antibody response to *B. canis* in stray dogs cohabiting the same area as the elephant herd using commercial Dot-ELISA. We mainly investigated the infection by *B. canis* in stray dogs because this pathogen is a predominant strain in dogs [34]. Importantly, the principle of this commercial kit is based on the detection of serum-specific antibody against *B. canis* associated with the rough LPS antigen, which is antigenically different from the sLPS. The result from Dot-ELISA revealed low positivity against *B. canis* (12.5%) in stray dogs. We supposed that *B. canis* infection in stray dogs in the present study was caused by natural infection. Interestingly, stray dogs are more likely to have a higher level of *B. canis* seropositivity compared to owned dogs [35]. However, in the absence of the full epidemiological information, it is difficult to make conclusions between seropositive dogs and the potential for Asian elephant exposure, but future investigation could clarify the risk potential.

## Conclusion

The serological screening and surveillance for possible *Brucella* infection is necessary and remains the major diagnostic tool for regular disease monitoring in Asian elephant. Moreover, an initial screening of brucellosis could be performed by conventional serological test, such as RBPT and EDTA-TAT followed by a more specific iELISA. However, the investigations using modified serological tests in wild animals should be further validated if it is possible, together with comparing with bacterial isolation and identification or gold standard test, to establish an appropriate brucellosis surveillance program in Asian elephants. Moreover, the investigation of possible rough *Brucella* infection in Asian elephant should be significantly considered.

## Authors’ Contributions

SN created the research and experimental design, performed the laboratory experiment and data analysis, and wrote the manuscript. TA, WN, and NT performed the sample collection and helped in the laboratory work. All authors read and approved the final manuscript.
